# Extended Resections for Advanced Gallbladder Cancer: Results from a Nationwide Cohort Study

**DOI:** 10.1245/s10434-020-08858-z

**Published:** 2020-07-21

**Authors:** H. Kuipers, E. A. J. de Savornin Lohman, M. van Dooren, A. E. Braat, F. Daams, R. van Dam, J. I. Erdmann, J. Hagendoorn, F. J. H. Hoogwater, B. Groot Koerkamp, T. M. van Gulik, P. R. de Reuver, M. T. de Boer

**Affiliations:** 1 Department of Surgery, Section Hepato-Pancreato-Biliary Surgery and Liver Transplantation, University of Groningen, University Medical Center Groningen, Groningen, The Netherlands; 2 Department of Surgery, Radboud University Medical Center, Nijmegen, The Netherlands; 3 Department of Surgery, Leiden University Medical Center, Leiden, The Netherlands; 4 Department of Surgery, Amsterdam University Medical Centers, University of Amsterdam, Amsterdam, The Netherlands; 5 Department of Surgery, Maastricht University Medical Center, Maastricht, The Netherlands; 6 Department of Surgery, Utrecht University Medical Center, Utrecht, The Netherlands; 7 Department of Surgery, St. Antonius Hospital, Nieuwegein, The Netherlands; 8 Department of Surgery, Erasmus Medical Center, Rotterdam, The Netherlands

## Abstract

**Background:**

Extended resections (i.e., major hepatectomy and/or pancreatoduodenectomy) are rarely performed for gallbladder cancer (GBC) because outcomes remain inconclusive. Data regarding extended resections from Western centers are sparse. This Dutch, multicenter cohort study analyzed the outcomes of patients who underwent extended resections for locally advanced GBC.

**Methods:**

Patients with GBC who underwent extended resection with curative intent between January 2000 and September 2018 were identified from the Netherlands Cancer Registry. Extended resection was defined as a major hepatectomy (resection of ≥ 3 liver segments), a pancreatoduodenectomy, or both. Treatment and survival data were obtained. Postoperative morbidity, mortality, survival, and characteristics of short- and long-term survivors were assessed.

**Results:**

The study included 33 patients. For 16 of the patients, R0 resection margins were achieved. Major postoperative complications (Clavien Dindo ≥ 3A) occurred for 19 patients, and 4 patients experienced postoperative mortality within 90 days. Recurrence occurred for 24 patients. The median overall survival (OS) was 12.8 months (95% confidence interval, 6.5–19.0 months). A 2-year survival period was achieved for 10 patients (30%) and a 5-year survival period for 5 patients (15%). Common bile duct, liver, perineural and perivascular invasion and jaundice were associated with reduced survival. All three recurrence-free patients had R0 resection margins and no liver invasion.

**Conclusion:**

The median OS after extended resections for advanced GBC was 12.8 months in this cohort. Although postoperative morbidity and mortality were significant, long-term survival (≥ 2 years) was achieved in a subset of patients. Therefore, GBC requiring major surgery does not preclude long-term survival, and a subgroup of patients benefit from surgery.

Gallbladder cancer (GBC) is a rare tumor. Worldwide incidence rates are fewer than 2 per 100,000, with significant geographic variation.^1^,^2^ Nevertheless, it is the most common biliary tumor.^3^,^4^ Gallbladder cancer is characterized by locally aggressive behavior and early spread to regional lymph nodes.^1^ Timely diagnosis of GBC is difficult due to the late, nonspecific symptoms and a tendency for early metastatic spread.^5^ As a result, GBC is diagnosed at an advanced stage in the majority of cases.^6^–^8^

Complete surgical resection is the only curative treatment.^9^ The majority of long-term survivors are patients with GBC diagnosed incidentally after cholecystectomy for presumed benign gallbladder disease. Only 10% to 20% of tumors diagnosed preoperatively, are amenable to resection at presentation, and the prognosis after resection remains unfavorable.^10^–^14^ Patients with T4 disease, even after radical resection, have a median overall survival (OS) period of only 11 months.^14^

To achieve resection with tumor-free margins, extended resections such as major hepatectomy, pancreatoduodenectomy (PD), and even hepatopancreatoduodenectomy have been performed for advanced tumors.^15^–^17^ However, extended resections are associated with significant postoperative morbidity and mortality (> 50%), whereas the benefit in terms of survival remains unclear.^15^ Moreover, almost all published studies are single-center series from non-Western countries. Only two Western studies were published more than one decade ago.^15^,^18^ Nationwide data are essential for light to be shed on actual clinical outcomes for GBC patients.

This study aimed to analyze the results of extended resections for patients with advanced GBC in a Dutch multicenter nationwide study, to determine postoperative morbidity, mortality, and survival, and to identify factors associated with short- and long-term survival.

## Methods

The study was approved by the Medical Ethics Committee of the Arnhem-Nijmegen region (METc no. 2017-3912) and conducted according to Strengthening the Reporting of Observational Studies in Epidemiology (STROBE) guidelines for observational cohort studies.^19^ Patients were identified from the Netherlands Cancer Registry (NCR). The NCR contains data on all newly diagnosed malignancies including year of diagnosis, patient age and gender, tumor characteristics (tumor-node-metastasis [TNM] stage), patient identification number, and treatment hospital. The data from the NCR are based on data from the automated pathologic archive (PALGA), the nationwide network, and the registry of histo- and cytopathology in The Netherlands, supplemented by data from the National Archive of Hospital Discharge Diagnosis.^20^ Patients treated for GBC in any tertiary referral center in The Netherlands were included in a national, retrospective database.

### Patient Selection and Variable Definitions

Patients with histopathologically proven GBC who underwent extended resection were included in the current study. Extended resection was defined as a major hepatectomy (resection of ≥ 3 liver segments), a PD, or both, with or without en bloc resection of adjacent organs (duodenum, colon, or stomach) with curative intent. The study excluded patients who underwent a simple cholecystectomy, a resection or re-resection of the gallbladder bed, a minor hepatectomy (< 3 segments), and/or lymph node dissection along the hepatoduodenal ligament without associated major liver resection. Patients with incidentally diagnosed GBC (i.e., during or after cholecystectomy for presumed benign disease) also were excluded.

Data on patient characteristics, preoperative bilirubin and carbohydrate antigen 19-9 (CA19-9), operative characteristics, tumor characteristics, postoperative morbidity and mortality, recurrence, and OS were obtained from the medical records. Tumor staging was reported according to the American Joint Committee on Cancer (AJCC) staging system.^21^ Resection margins were classified as R0 (margin distance to tumor ≥ 1 mm) and R1 (micro- or macroscopically positive margin). Intraoperative hemorrhage was defined as intraoperative blood loss of at least 1000 ml or blood loss requiring a blood transfusion intraoperatively.

Data on postoperative complications were determined according to the Clavien–Dindo classification system and included complications up to 90 days after surgery.^22^ Major complications were defined as Clavien–Dindo grade 3A or higher. Postoperative mortality was defined as death due to any cause within 90 days postoperatively.

Determination of OS included deaths from any cause. Short-term survival was defined as survival up to 6 months, including postoperative mortality. Long-term survival was defined as survival 2 years or longer. Disease-free survival was defined as the number of months from extended resection to the date of recurrence or the date of the last follow-up assessment. Adjuvant therapy was administered only in a clinical trial setting because it was not considered standard of care during the study period.

### Statistical Analysis

Continuous variables are presented as median (interquartile range), and categorical data are presented as number (%). Survival was reported using Kaplan–Meier methods, and differences in survival were analyzed using the log-rank test. All *p* values of 0.05 or lower were considered statistically significant. All statistical analyses were performed using SPSS Statistics for Windows, version 23.0 (IBM Corporation, Armonk, NY, USA).

## Results

### Patient Characteristics

Between January 2000 and September 2018, 289 patients underwent a surgical resection for pre- or postoperatively diagnosed GBC with curative intent in the participating centers. During this period, 33 patients with pre-or intraoperatively diagnosed GBC (11%) underwent an extended resection and were included in the study.

The cohort consisted of 13 men (39%) and 20 women (61%). The median age at the time of diagnosis was 64 years (interquartile range [IQR], 57.0–68.5 years) (Table 1). The presenting symptoms were jaundice (*n* = 21), abdominal pain (*n* = 16), nausea (*n* = 8), weight loss (*n* = 7), discolored defecation (*n* = 3), fever (*n* = 1), back pain (*n* = 1), and liver enzyme disorders (*n* = 1). At presentation, CA19-9 was tested in 12 patients and showed a median value of 542 kU/l (IQR, 87–3500 kU/l). At presentation, bilirubin levels were available for 19 patients, with a median value of 94 µmol/l (IQR, 12–159 µmol/l). None of the patients were Asian or had a diagnosis of primary sclerosing cholangitis (PSC).

**Table 1:** Patient and operative characteristics of gallbladder cancer patients who underwent extended resection

	Total (*n* = 33) *n*	%
Median age: years (IQR)	64	(57–69)
*Gender*		
Female	20	(61)
Male	13	(39)
*ASA classification*^*a*^		
1	5	(20)
2	14	(56)
3	6	(24)
*Preoperative biliary drainage*		
No	13	(39)
Yes	20	(61)
*PVE performed*		
No	28	(85)
Yes	5	(15)
*Type of surgery*		
Left hemihepatectomy	1	(3)
Extended right hemihepatectomy	7	(21)
Right hemihepatectomy	11	(33)
Right hemihepatectomy + PD	2	(6)
PD + wedge	11	(33)
PD + segments 4 and 5	1	(3)
*Portal vein reconstruction*		
No	23	(70)
Yes	10	(30)
*Postoperative complications*		
No	5	(15)
Yes, < CD3	9	(27)
Yes, ≥ CD3	19	(58)

*IQR* interquartile range, *ASA* American society of anesthesiologists, *PVE* portal vein embolization, *PD* pancreatoduodenectomy, *CD* Clavien–Dindo classification system

^a^Eight missing values

### Preoperative Workup and Treatment

The preoperative workup consisted of ultrasonography (US) (*n* = 25), endoscopic US (*n* = 5), computed tomography (CT) (*n* = 33), magnetic resonance imaging (MRI) (*n* = 20), and positron emission tomography (PET)-CT (*n* = 5). Preoperative imaging for 30 patients (91%) showed adjacent organ invasion into the extrahepatic bile ducts (*n* = 22, 67%), liver (*n* = 16, 49%), pancreas (*n* = 7, 21%), or duodenum (*n* = 1, 3%). Preoperative biliary drainage was performed for 20 patients, and all 20 patients (61%) underwent endoscopic retrograde cholangiopancreatography. For two patients (6%), an additional percutaneous transhepatic cholangiography was required. For eight patients (24%), a diagnostic laparoscopy was performed. A portal vein embolization was performed for five patients (15%). None of the patients received neoadjuvant chemotherapy (NACT).

The indications for extended resection were suspicion for GBC, cholangiocarcinoma (CCA), or pancreatic cancer with invasion of adjacent organs. The main indications for major hepatectomies were a suspicion of hilar CCA (*n* = 4) or GBC with liver involvement, discovered on preoperative imaging (*n* = 11) or intraoperatively (*n* = 4). One of these patients had a clear indication for a major hepatectomy instead of a minor hepatectomy, which could not be obsoleted retrospectively. The main indications for a PD were suspicion for distal CCA (*n* = 2), suspicion for pancreatic cancer (*n* = 2), GBC with pancreatic involvement or suspicious lymph nodes around the pancreas preoperatively (*n* = 5), and GBC with involvement of the duodenum intraoperatively (*n* = 3). The indication for hepatopancreatoduodenectomy was GBC with involvement of the liver and extension into the distal bile duct and portal vein shown on preoperative imaging (*n* = 1) as well as GBC with involvement of the liver and intraoperative suspected lymph node invasion around the pancreas requiring resection of the pancreatic head (*n* = 1).

### Operative Characteristics

The operative characteristics of the entire cohort are presented in Table 1. The surgical procedures consisted of right hepatectomy (*n* = 11), extended right hepatectomy (*n* = 7), PD with wedge resection (*n* = 11), PD with concurrent segment 4 and 5 resection (*n* = 1) and left hepatectomy (*n* = 1), and PD combined with right hepatectomy (*n* = 2). Additionally, the colon was partially resected due to intraoperative involvement in two cases, and the ovaries were resected in one case. All the patients underwent a lymph node dissection and resection of the common bile duct (CBD). The portal vein was reconstructed in 10 patients (30%).

Intraoperative complications occurred for five patients (15%), consisting of hemorrhage in five patients and an additional systemic inflammatory response syndrome (SIRS) in one patient. All the intraoperative complications occurred for patients who underwent a major hepatectomy (Table 2).

**Table 2:** Serious (≥ CD3a) postoperative complications within 90 days after extended resections in gallbladder cancer patients

	Major hepatectomy (*n* = 19) *n* (%)	PD (*n* = 12) *n* (%)	Major hepatectomy + PD (*n* = 2) *n* (%)
Postoperative complications ≥ CD3	11 (58)	7 (58)	1 (50)
Intraabdominal abscess	1	3	0
Ascites	4	0	0
Abdominal hemorrhage	0	3	1
Anastomotic leakage	1	3	0
Pancreatic fistula	1	0	0
Respiratory	3	1	1
Cardiac	1	0	0
Liver failure	1	1	0
Sepsis/SIRS/systemic shock	4	3	0
Other	2	2	1
Postoperative mortality	3 (16)	1 (8)	0 (0)

*PD* pancreatoduodenectomy, *CD* Clavien–Dindo classification system, *SIRS* systemic inflammatory response syndrome

### Tumor Characteristics

Histopathologic analysis showed tumor-free margins in 16 patients (49%; Table 3). Histology showed adenocarcinoma (*n* = 29), squamous-cell carcinoma (*n* = 1), and adenosquamous carcinoma (*n* = 1). The histopathologic subtype was not described for two patients. Tumor differentiation grade, reported for 22 patients, was good in 6 patients (27%), moderate in 9 patients (41%), and poor in 7 patients (32%). Perineural invasion was found in 24 patients (73%) and perivascular invasion in 16 patients (49%).

**Table 3:** Pathologic characteristics of gallbladder cancer patients after extended resections

	Total (*n* = 33) *n*	(%)
*pT*^*a*^		
T1	0	(0)
T2	4	(13)
T3	18	(55)
T4	10	(31)
*pN*		
N0	13	(39)
N1	11	(33)
N2	9	(27)
*pM*^*b*^		
M0	22	(71)
M1	9	(29)
*Resection margin*		
R0	16	(49)
R1	17	(52)
*Differentiation grade*^*c*^		
Good	6	(27)
Moderate	9	(41)
Poor	7	(32)
*Perineural invasion*		
No	9	(27)
Yes	24	(73)
*Vascular invasion*		
No	17	(52)
Yes	16	(49)
*Liver invasion*		
No	12	(36)
Yes	21	(64)
*Histology*^*b*^		
Adenocarcinoma	29	(94)
Squamous-cell carcinoma	1	(3)
Adenosquamous carcinoma	1	(3)

^a^One missing value

^b^Two missing values

^c^Eleven missing values

### Postoperative Morbidity and Mortality

Major postoperative complications within 90 days occurred for 19 patients (58%), as described in Table 2. Four postoperative deaths (12%) occurred, two due to sepsis (because of liver failure or anastomotic leakage), one due to liver failure after portal vein thrombosis, and one due to aspiration and hypoxia.

### Adjuvant Treatment, Follow-Up Evaluation, and Survival

Two patients received adjuvant chemotherapy (gemcitabine and cisplatin for 1 patient and capecitabin for 1 patient), and five patients received chemotherapy with palliative intent at the time of recurrence. Recurrence occurred for 24 patients (73%) after a median disease-free interval of 11 months (95% confidence interval [CI], 6.4–15.7 months). Imaging during follow-up assessment showed recurrence locally (*n* = 13), on the peritoneum (*n* = 10), in the liver (*n* = 9), in the lungs (*n* = 1) and at other locations (*n* = 7). All mortality was disease-related (i.e., due to postoperative complications, progression, or recurrence).

**Fig. 1:**
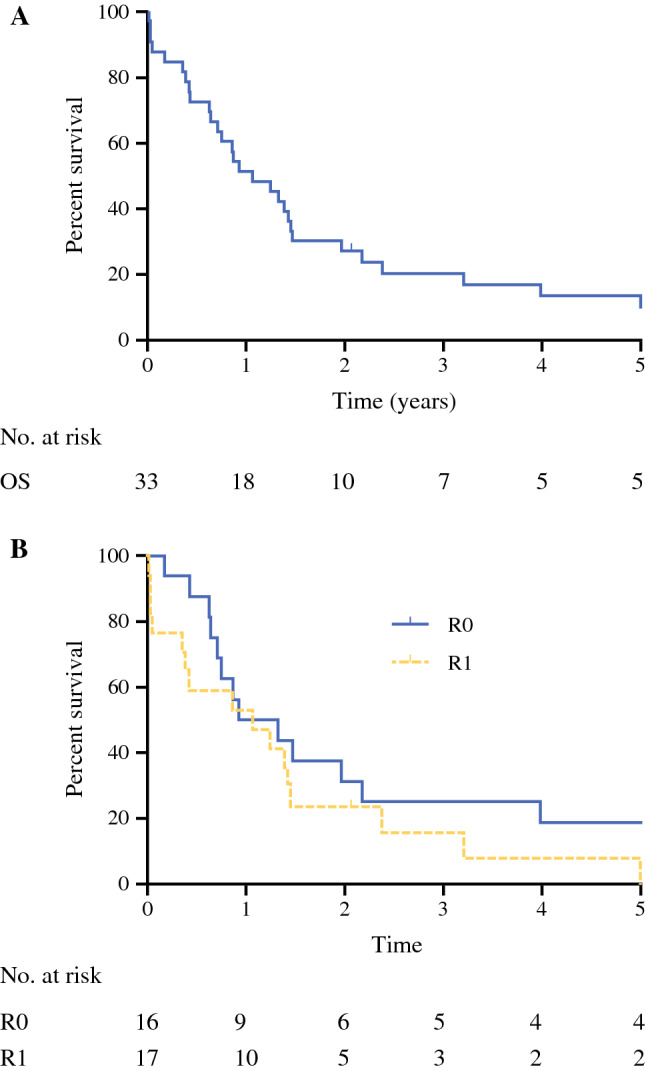
Overall survival (OS) in years

The median OS was 12.8 months (95% CI, 6.5–19.0 months; Fig. 1a) after a median follow-up period of 13 months. The median OS, excluding postoperative mortality, was 15.9 months (95% CI, 9.1–22.7 months), and the median disease-free survival was 10.1 months (95% CI, 4.5–15.8 months).

No significant survival difference was found between R0 and R1 resections. The median OS was 11.1 months for the R0 patients versus 12.8 for the patients with a R1 resection (*p* = 0.203; Fig. 1B). Three patients were still alive without signs of disease at the time of this study (median follow-up period, 97 months).

The characteristics of the short-term survivors (≤ 6 months; *n* = 9) and the long-term survivors (≥ 2 years; *n* = 10) are reported in Tables 4 and 5, respectively. When the patients who died of postoperative complications were excluded, eight of nine short-term survivors showed jaundice at the time of presentation, and all the patients showed perineural and perivascular invasion, invasion of the liver parenchyma, and CBD in the histopathologic analysis. All the long-term survivors without recurrence (*n* = 3) showed tumor-free resection margins without signs of perivascular invasion or liver invasion in the histopathologic analysis. One long-term survivor without recurrence received adjuvant chemotherapy.

**Table 4:** Characteristics of short-term survivors (≤ 6 months)

Age (years),^a^ gender, ASA	Jaundice^a^	Procedure	pTNM R status	Perineural invasion	Vascular invasion	Liver invasion	CBD invasion	Follow-up (days)	Cause of death
66, M, 3	Yes	RH	T4N0M0 R0	Yes	Yes	Yes	No	6	Postop comp
77, M, NA	Yes	RH	T3N1M0 R1	Yes	Yes	Yes	Yes	10	Postop comp
73, M, 2	Yes	RH	T3N0Mx R1	Yes	No	Yes	Yes	10	Postop comp
64, F, NA	Yes	PD	T4N1M1 R1	Yes	Yes	Yes	Yes	17	Postop comp
64, F, 2	Yes	PD	T3N2M1 R0	Yes	Yes	Yes	Yes	63	Progression
70, F, 3	No	RH	T4N1M0 R1	Yes	Yes	Yes	Yes	130	Progression
58, F, 3	Yes	PD	T4N2M1 R1	Yes	Yes	Yes	Yes	141	Progression
64, M, 2	Yes	Ext RH	T3N1M0 R1	Yes	Yes	Yes	Yes	155	Progression
67, M, NA	Yes	Ext RH	T3N0M0 R0	Yes	Yes	Yes	Yes	157	Recurrence

*ASA* American society of anesthesiologists, *pTNM* pathologic tumor-node metastasis, *CBD* common bile duct, *M* male, *RH* right hemihepatectomy, *Postop comp* postoperative complications, *F* female, *PD* pancreatoduodenectomy, *Ext RH* extended right hemihepatectomy, *NA* not available

^a^At presentation

**Table 5:** Characteristics of long-term survivors (≥ 24 months)

Age (years),^a^ gender, ASA	Jaundice^a^	Procedure	pTNM R status	Perineural invasion	Vascular invasion	Liver invasion	CBD invasion	Follow-up (months)	Status
65, M, 2	Yes	PD	T3N0M0 R0	No	No	Yes	No	24	Deceased, recurrence
63, M, 2	Yes	RH	T2N0M0 R1	Yes	Yes	No	No	24	Alive, with recurrence
67, F, 2	No	RH	T4N1M0 R0	Yes	No	No	No	27	Deceased, recurrence
79, F, 2	No	PD	T2N2M0 R1	No	Yes	No	No	29	Deceased, recurrence
63, M, 3	Yes	PD	T4N1M0 R1	Yes	No	No	No	39	Deceased, recurrence
46, M, 2	Yes	RH + PD	T3N1M0 R0	Yes	Yes	Yes	Yes	49	Deceased, recurrence
69, F, 2	Yes	RH + PD	T4N2M1 R1	Yes	No	No	No	71	Deceased, recurrence
65, M, 3	No	RH	T3N0M0 R0	Yes	No	No	Yes	86	Alive, no recurrence
53, F, 1	No	RH	T2N2M0 R0	No	No	No	No	91	Alive, no recurrence
68, F, 2	No	PD	T3N0M0 R0	No	No	No	No	119	Alive, no recurrence

*ASA* American Society of Anesthesiologists, *pTNM* pathologic tumor-node metastasis, *CBD* common bile duct, *M* male, *PD* pancreatoduodenectomy, *RH* right hemihepatectomy, *F* female

^a^At presentation

## Discussion

This national, retrospective cohort analysis is the first Western study in the past decade to investigate the outcomes of extended resections for GBC. This series showed that major hepatectomies and PDs rarely are performed for advanced GBC in The Netherlands. Despite a reported median disease-free survival of 10.1 months and a median OS of 12.8 months, 21% of the patients survived beyond 3 years. Major postoperative complications occurred for 58% of the patients and postoperative mortality for 12% of the patients. In 49% of the patients, R0 resection margins were achieved.

In a study from the Memorial Sloan Kettering Cancer Center,^15^ comparable mortality rates were described, with a postoperative mortality rate of 14% (5 of 36 patients) for major hepatectomies. Recurrence occurred for 24 patients (73%). In that study, R0 resection margins were achieved for 91% of the patients, and the 5-year survival rate was 27%. Nevertheless, in that study, some patients without evidence of inflow involvement underwent empiric major hepatectomy, whereas in our study all the patients except one were suspected of tumor extension to other organs, necessitating extended resection for tumor-free margins.

The value of extended surgery for advanced GBC remains questionable. Results from previous studies investigating patients undergoing hepatopancreaticoduodenectomy show virtually no survival beyond 2 years and a R0 resection rate of only 20%.^23^,^24^ In our cohort, the 2-year survival rate was 30% and the 5-year survival rate was 12%. The patients with an R0 resection (achieved for 49%) had a 5-year survival rate of 19%. Multivariable analysis in a previous study by Fong et al.^25^ showed that the extent of liver resection did not influence long-term survival. Another study argued that wedge resection is to be reserved for patients with minimal liver invasion and that extensive liver resections should be performed for patients who have advanced tumors with extensive liver invasion or hepatic-hilar-type tumors.^26^ Unfortunately, due to low numbers of patients, we could not study the association between extent of liver invasion, resection, and survival. However, it is known from a population-based study that the 1-year survival rate for patients with unresected, advanced GBC is less than 10%, versus a 1-year survival of 55% and a median OS of 12.8 months in our cohort including patients who died of surgery-related complications.^27^ The median OS of all patients who had unresected GBC treated with palliative chemotherapy was 6.4 months in our nationwide cohort (unpublished results). Moreover, in the ABC-02 trial (gemcitabine + cisplatin vs gemcitabine alone for unresected biliary tract cancer), no survivors beyond 3 years were reported, whereas our cohort had a 3-year survival rate of 20%.^28^

Although no significant survival difference between R0 and R1 patients was observed, all three long-term survivors without recurrence clearly had R0 resection margins. The lack of a statistically significant difference therefore was most likely caused by our small sample size. Other explanations include possible per-centra and over-time differences in R0 or R1 resection margin criteria as well as differences in experience and quality of pathologists.

Identification of prognostic factors is vital for adequate patient selection. Our results show that except for a short-term survivor, all the patients were jaundiced at presentation, a factor known to have a negative influence on survival.^29^ Additionally, all the short-term survivors had a pT3 or pT4 stage tumor, and the majority had positive lymph nodes. In contrast, few long-term survivors had a high T stage and positive lymph nodes. Except for the patients who died of postoperative complications, all the short-term survivors had both perivascular invasion and invasion of the liver parenchyma. These factors also were associated with poor survival in other studies.^24^,^30^,^31^

In-depth preoperative assessment using imaging techniques such as contrast-enhanced MRI may identify patients with smaller, localized tumors amenable to resection.^32^ The use of PET scans and PET-CT scans might be especially helpful in detecting unsuspected metastasis.^33^–^35^

Although our cohort likely comprised a highly selected subgroup of patients fit to undergo extensive surgery with no suspicion for metastasis, our results demonstrate that for these patients, long-term survival after extended surgery is possible. However, the high morbidity and mortality rates associated with extensive liver surgery need to be weighed against the apparent survival benefit. Postoperative quality of life (QoL) must be taken into account when extended resection is considered. Unfortunately, due to the retrospective nature of this study, we were not able to assess QoL by using questionnaires.

Identification of the correct tumor type and location preoperatively may be difficult in GBC. In 12 of 33 cases of our series, CCA or pancreas carcinoma was suspected instead of GBC preoperatively. Infiltration by tumor or inflammation in surrounding tissues (i.e., pancreas, hilum or extrahepatic bile ducts) makes identification of primary tumor location on preoperative imaging challenging. Moreover, differentiating malignant invasion in adjacent organs from inflammation is difficult. In a study from Memorial Sloan Kettering Cancer Center, a subgroup of patients required resection of adjacent organs due to tumor adhesion. Definitive histopathology showed tumor invasion in only half of these patients.^15^

Results from the recently published BILCAP trial suggest that adjuvant capecitabine can improve OS for patients with resected biliary tract cancer.^36^ Currently, adjuvant or neoadjuvant chemotherapy is not considered standard of care in The Netherlands, as reflected by the small number of patients in our cohort who received chemotherapy.^37^ However, one of three long-term survivors without recurrence received adjuvant capecitabine, providing additional support for the use of adjuvant chemotherapy.

Moreover, a recent article reported significantly lower recurrence rates and better OS for patients receiving adjuvant chemoradiation therapy versus surgery alone for resected GBC. Notably, this benefit was observed only for patients with positive lymph node status, and patients with N0 disease did not appear to benefit from adjuvant therapy.^38^ Therefore, administration of adjuvant chemoradiation might be helpful for pN1/2 patients fit to undergo adjuvant therapy.

A recent systematic review stated that although favorable tumor response and increased resectability rates have been reported after NACT, the evidence currently is insufficient to support its routine use.^39^ However, the authors also concluded that future randomized trials should be conducted to investigate the role of NACT in advanced GBC. Because radical resection seems to be the only way long-term survival can be achieved, NACT before resection for locally advanced gallbladder cancer patients (e.g., the patients in our cohort) may further improve outcomes.

Our study had several limitations. First, the small number of patients made it impossible to draw statistical conclusions about prognostic factors for prolonged survival after extended resections for GBC Second, due to the retrospective nature of the study, a large amount of data were missing. Unfortunately, prospective research is logistically challenging due to the low incidence of this type of tumor. Future international collaborative studies should include a larger cohort of patients, preferably based on prospective data collection.

In conclusion, median OS after major resection for advanced GBC in our cohort was 12.8 months, and 10 patients survived longer than 2 years. Jaundice at the time of presentation, perineural and perivascular invasion, positive lymph nodes, and invasion of the liver parenchyma and CBD demonstrated via histopathologic examination were present in patients with poor survival. Because major postoperative complications were frequent and postoperative mortality occurred in 12% of the patients, the prognosis for these patients was extremely poor if no surgery was performed. Therefore, extended resections for patients with locally advanced GBC should be considered if the morbidity and mortality rates are acceptable for the patient compared with the presumed benefit in terms of survival and QoL.
